# Half-Yearly Report of Cases in Midwifery, Which Have Occurred in the Northern District of the London and Southwark Midwifery Institution

**Published:** 1831-08

**Authors:** C. Waller

**Affiliations:** Consulting Accoucheur to the above Institution, and Lecturer on Midwifery at the Medical School, 58, Aldersgate street.


### MIDWIFERY.

*Half-yearly Report of Cases in Midwifery, which have occurred in the Northern District of the London and Southwark Midwifery Institution.* By C. Waller, Esq. Consulting Accoucheur to the above Institution, and Lecturer on Midwifery at the Medical School, 58, Aldersgate street.

| 1831. | Number of Women delivered. | Sex of Children. | | Born Alive. | Stillborn. | Presentation. |
|---|---|---|---|---|---|---|
| | | Males. | Females. | | | |
| January... | 35 | 22 | 13 | 31 | 4 | 32 Natural<br>1 Foot<br>1 Premature<br>1 Face to Pubis |
| February . | 27 | 16 | 11 | 25 | 2 | Natural |
| March ... | 35 | 18 | 17 | 34 | 1 | 34 Natural<br>1 Breech |
| April .... | 29 | 12 | 17 | 25 | 4 | 28 Natural<br>1 Breech<br>1 Premature |
| May ..... . | 41 | 20 | 21 | 37 | 4 | 39 Natural<br>1 Face to Pubis<br>1 Foot<br>1 Premature |
| June ..... | 38 | 24 | 15 | 39 | 0 | 37 Natural<br>1 Face to Pubis<br>1 Case of Twins |
| Total.... | 205 | 112 | 94 | 191 | 15 | |

Nothing very particular has occurred in our institution during the past six months; and when we consider the great tendency there has been, of late, to inflammatory diseases, it seems rather extraordinary that the parturient female should have escaped, especially as our patients are frequently residing in close and unhealthy situations.

A few cases of slight hemorrhage have occurred, requiring only the usual applications for its suppression.

Several very obstinate cases of diarrhœa have also been witnessed: in one instance the symptoms were sufficiently severe to warrant a suspicion that ulceration of the inner membrane of the bowels had taken place, the patient complaining of violent griping pains in the abdomen, attended with a distressing degree of tenesmus. The evacuations consisted of a purulent-looking slime, mixed with blood; and at times pure blood was discharged.

The most powerful astringents appeared to give the greatest relief in these cases, more particularly the sulphate of copper, with opium, beginning with a quarter or half a grain of the former, combined with half a grain of the latter, and repeating the dose every four hours. This plan of treatment was, if my recollection serves me, first brought before the public by Dr. Sutton, of Greenwich, in a paper published about twelve years ago, in the London Medical and Physical Journal. This remedy succeeded in arresting one very formidable case, which lately occurred in my private practice.

I was called to this patient, who had been in labour for three days, and was obliged to have recourse to instruments: she was in such a state of exhaustion, that I feared no rally would take place. She, however, revived; and on the third day a dose of castor oil was given her: this not operating, and her abdomen getting very much distended, I ordered her an enema composed of gruel, with an ounce and a half of oil of turpentine. Her bowels were freely relieved, but a state of diarrhœa supervened, which resisted the ordinary remedial agents. To so low a state was she reduced, that the fæces passed involuntary: she was at length completely cured by the sulphate of copper, with opium. She, of course, remained for a long period in a very debilitated condition.

A case or two of simple fever, without any abdominal affection, may be reported, which, although tedious, yielded to the ordinary method of treating febrile affections. A case of this kind, which occurred in private practice, to which I was called in consultation, proved fatal: in this instance, however, the lady had lost a very large quantity of blood after the birth of the placenta.

*Bartholomew Close; July* 1831.

